# Controlled processing during sequencing

**DOI:** 10.3389/fnhum.2015.00599

**Published:** 2015-10-29

**Authors:** Malathi Thothathiri, Michelle Rattinger

**Affiliations:** Department of Speech and Hearing Science, The George Washington University, WashingtonDC, USA

**Keywords:** sequencing, control, language, BA 44/6, prefrontal cortex, premotor cortex, inferior frontal junction

## Abstract

Longstanding evidence has identified a role for the frontal cortex in sequencing within both linguistic and non-linguistic domains. More recently, neuropsychological studies have suggested a specific role for the left premotor-prefrontal junction (BA 44/6) in selection between competing alternatives during sequencing. In this study, we used neuroimaging with healthy adults to confirm and extend knowledge about the neural correlates of sequencing. Participants reproduced visually presented sequences of syllables and words using manual button presses. Items in the sequence were presented either consecutively or concurrently. Concurrent presentation is known to trigger the planning of multiple responses, which might compete with one another. Therefore, we hypothesized that regions involved in controlled processing would show greater recruitment during the concurrent than the consecutive condition. Whole-brain analysis showed concurrent > consecutive activation in sensory, motor and somatosensory cortices and notably also in rostral-dorsal anterior cingulate cortex. Region of interest analyses showed increased activation within left BA 44/6 and correlation between this region’s activation and behavioral response times. Functional connectivity analysis revealed increased connectivity between left BA 44/6 and the posterior lobe of the cerebellum during the concurrent than the consecutive condition. These results corroborate recent evidence and demonstrate the involvement of BA 44/6 and other control regions when ordering co-activated representations.

## Introduction

Temporal sequencing or the ability to order representations over time is an important component of language and other cognitive functions. For example, during language production, a speaker must sequence words and sounds in order to express a coherent sentence. Similarly, planning any multi-step action—whether it be concrete (e.g., brushing one’s teeth) or more abstract (e.g., an annual vacation)—involves accessing the individual steps and assembling them into a sequence.^1^ Neuropsychological and neuroimaging studies indicate a role for the frontal cortex in sequencing within language and other domains (Neuropsychological: Lepage and Richer, 1996; Zanini et al., 2002; Papeo et al., 2015; Neuroimaging: Gelfand and Bookheimer, 2003; Grewe et al., 2006; Uddén et al., 2008; Bahlmann et al., 2012). We have previously demonstrated a link between the sequencing of phonological representations and the left premotor-prefrontal junction, in particular, in patients diagnosed with aphasia (Thothathiri et al., 2010, 2012). In the present study, we used neuroimaging in healthy adults to seek convergent evidence and examine the role of this junction as well as other areas during temporal sequencing.

Dysfunction of the frontal lobe has been linked to sequencing deficits in different neuropsychological populations (Lepage and Richer, 1996; Zanini et al., 2002; Papeo et al., 2015; inter alia). The results largely support the idea that frontal involvement in sequencing pertains to controlled processing^2^ rather than the representation of specific content. For example, Zanini et al. (2002) tested patients with frontal lesions on an action sequencing task wherein participants were asked to order the steps involved in action schemas (e.g., a visit to the doctor). The patients were successful in producing and describing the individual steps, suggesting that their action knowledge was not impaired. However, they were impaired in ordering the steps, which the authors interpreted as resulting from an inability to reject wrong sequences and pick the correct sequence (Zanini et al., 2002). Papeo et al. (2015) showed a similar dissociation between producing/recognizing individual actions and the sequencing of those actions in individuals with amyotrophic lateral sclerosis (ALS). Given the link between ALS and frontal dysfunction, they endorsed the view that impairment to frontally mediated control might underlie these patients’ sequencing difficulties (Papeo et al., 2015). One specific explanation of sequencing deficits is that frontal patients are unable to resolve interference between multiple responses (Luria, 1966). Lepage and Richer (1996) tested this hypothesis explicitly using a sequence reproduction task where participants pressed keys corresponding to sequences of visually presented stimuli. The stimuli were presented either one at a time or all at once. In healthy participants and patients with non-frontal lesions, the simultaneous presentation of stimuli slowed response initiation but speeded up inter-response times, suggesting that subjects planned multiple responses ahead of time and obtained a benefit from such planning ahead. In contrast, patients with frontal excisions showed markedly slow sequence initiation but no speeding up of subsequent responses. The authors interpreted this pattern of performance as following from impairment in resolving inter-response interference (Lepage and Richer, 1996).

Studies in patients diagnosed with aphasia have led to similar conclusions regarding a role for the frontal cortex in resolving interference between responses and selecting the correct alternative during verbal production. For example, Robinson et al. (2005) have shown that patients with prefrontal damage and severely reduced spontaneous speech are impaired when they are asked to generate a verbal response under conditions of high competition but not low competition. Schwartz and Hodgson (2002) provided a detailed case study of a frontal patient who could produce individual words with high accuracy but nevertheless struggled to produce those words in the context of other words. Such deficits could be explained by an inability to resolve interference between multiple co-activated words (Schwartz and Hodgson, 2002).

The neuropsychological studies described above suggest a link between sequencing and the ability to select between competing responses. The results point broadly to the frontal cortex as a neural substrate. Neuroimaging studies have tied selection between competing alternatives to a more specific locus within the left ventrolateral prefrontal cortex (left VLPFC). This region is engaged during diverse tasks such as Stroop, verb generation, and proactive interference (Thompson-Schill et al., 1997, 1998; Nelson et al., 2009; Snyder et al., 2011). It is notable that while these standard cognitive control tasks all involve selection under competition (e.g., between the font color and the word in the Stroop task), few if any involve sequencing. Based on this evidence, Thothathiri et al. (2010) hypothesized that the left VLPFC might be involved in sequencing *because of* its role in selecting between competing alternatives. When multiple representations are activated at the same time, as in the case of items in a sequence, selection might be required for choosing the right representation for the right position in the sequence (“selection for position”: Thothathiri et al., 2010). To evaluate this hypothesis, these authors designed a multiword naming task that induced “selection for position” difficulty while keeping other task demands constant. Participants were primed to produce certain nouns in certain phrasal positions (e.g., ***glove*** *and duck*, ***glove*** *and carrot*) and were subsequently asked to either keep the nouns in the same position (e.g., ***glove*** *and whistle*) or switch to the alternate position (e.g., *whistle and* ***glove***). The researchers examined the performance of four aphasic patients who had damage to left VLPFC and predicted that the patients would have difficulty when asked to switch a noun from the primed to the unprimed position. Interestingly, not all left VLPFC patients showed the same pattern. Those with extensive damage to the posterior portion of left VLPFC—at the junction between Brodmann areas 44 and 6 (hereafter BA 44/6)-showed significantly greater impairment than the other frontal patients (Thothathiri et al., 2010). In a subsequent study (Thothathiri et al., 2012), the patients with damage to left BA 44/6 also showed greater difficulty in the sequence reproduction task described above (Lepage and Richer, 1996). Therefore, the authors suggested that damage to the left premotor-prefrontal junction in particular might cause difficulty in selection between co-activated representations, which in turn could lead to problems with sequencing and language production in individuals with aphasia (Thothathiri et al., 2010, 2012). Neuroimaging studies with healthy adults and neuropsychological results from dementia patients have also identified BA 44/6 as a possible locus of cognitive control and sequencing (Gelfand and Bookheimer, 2003; Brass et al., 2005; Grewe et al., 2006; Derrfuss et al., 2012; Schroeter et al., 2012).

In the present study, we sought convergent evidence on the neural correlates of sequencing, with a specific focus on left BA 44/6. To achieve this goal, we imaged healthy adults as they performed the sequence reproduction task used previously with frontal lesion patients (Lepage and Richer, 1996; Thothathiri et al., 2012). We conducted whole-brain analysis to identify the broad set of regions involved in sequencing, region of interest (ROI) analyses to examine neural activation as well as correlation between activation and behavioral performance within left BA 44/6, and functional connectivity analysis to explore the networks that are selectively activated when multiple responses are planned in conjunction. The stimuli to be sequenced were syllables and words. Because our primary interest was in phonological sequencing as it relates to language production and both stimulus types are phonological, we collapsed them together in all primary analyses. In secondary analyses, we looked for possible differences between the two stimulus types. Such differences might be expected if the sequencing of words automatically triggered semantic processing and used different neural substrates than the sequencing of syllables.

## Materials and Methods

### Participants

Fourteen right-handed native English speakers from the Washington, D.C. area completed the experiment (19–35 years. Mean = 22.8. nine female) and were paid $20 for their participation. Participants self-reported handedness and language history, and were screened for MRI safety. None reported previous head injury or psychiatric or neurological disorders. All gave consent under a protocol approved by the Institutional Review Board at The George Washington University.

### Procedure

**FIGURE 1:**
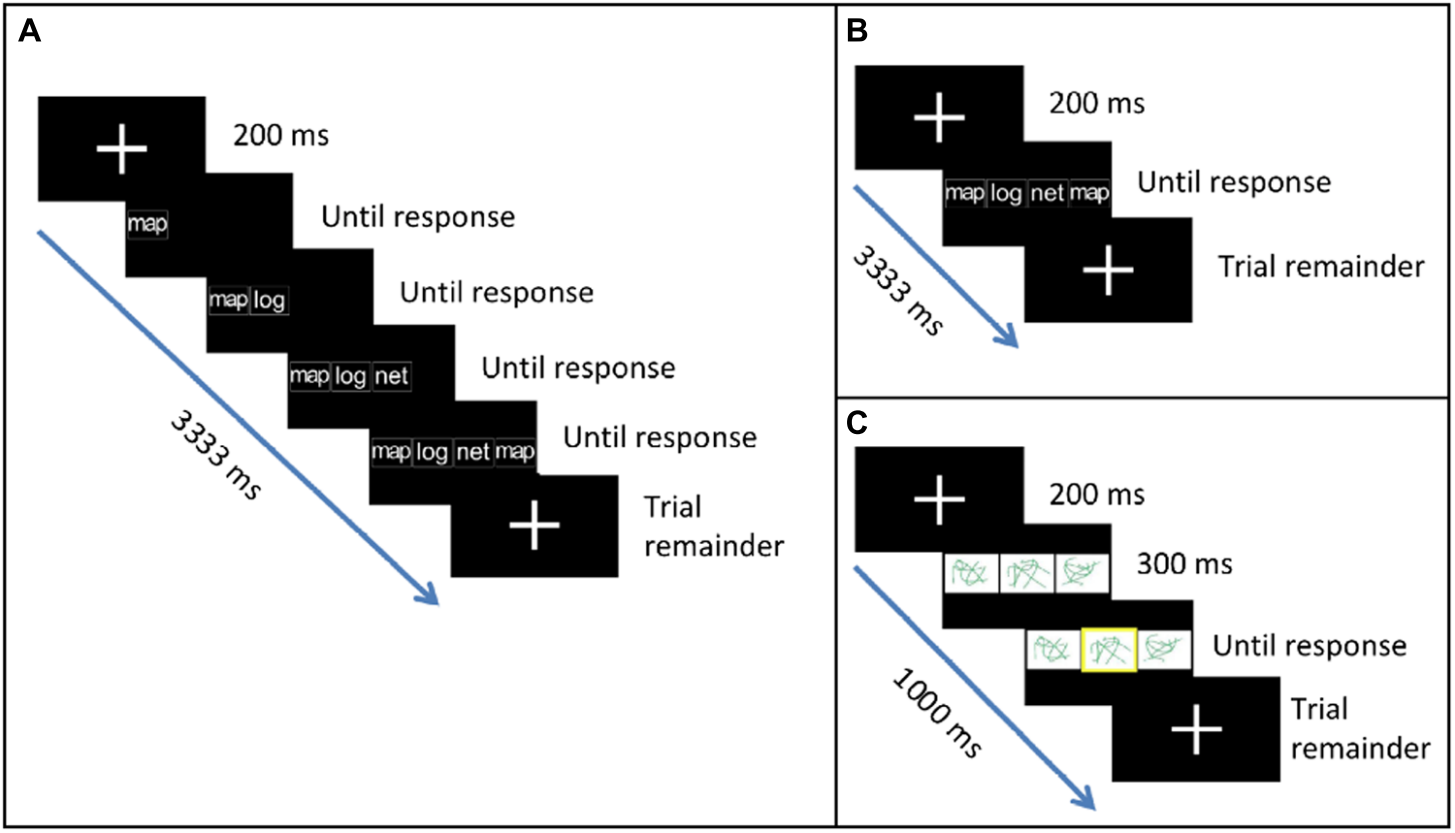
**Tasks used in the study.** **(A)** In the Consecutive task, participants replicated a sequence of syllables or words using button presses. Items in the sequence appeared one after another. **(B)** In the Concurrent task, items in the sequence appeared all together at once. **(C)** In the Baseline task, participants indicated which abstract line drawing was highlighted.

Participants completed three types of tasks (two critical, one baseline) presented in a block design. In the two critical experimental tasks, they saw four-item sequences of syllables (la, ma, na) or words (log, map, net) on the screen (e.g., ma la na ma; net map net log), and replicated each sequence from left to right using button presses. The items in each sequence appeared either consecutively (**Figure 1A**) or concurrently (**Figure 1B**). In the baseline task, they saw three abstract line drawings and indicated which drawing was highlighted (**Figure 1C**). Thus, the baseline task served as a control for basic visual and motor processing. The consecutive and the concurrent tasks both involved processing visually presented phonological items and choosing the corresponding motor responses. However, the concurrent task was expected to additionally involve the simultaneous processing of multiple items and the planning of ordered motor responses, thereby creating demands on sequencing and selection between competing alternatives.

Before scanning, participants underwent a short practice session where they were familiarized with the three tasks. In the scanner, they made responses using three buttons. For the critical tasks, the three buttons corresponded to the three unique syllables/words: button 1 for la/log, button 2 for ma/map, and button 3 for na/net. Thus, replicating the sequence “ma la na ma” required pressing buttons 2, 1, 3 and 2, in that order. For the baseline task, the three buttons corresponded to the locations of the line drawings (left, middle, and right). Thus, the correct response to the left picture being highlighted would be 1, etc. For all tasks, participants were asked to use their left thumb to press button 1 and their right thumb to press buttons 2 and 3.

Stimuli were presented using E-prime. Each trial began with a fixation cross (200 ms). In the consecutive condition, fixation was followed by the first item in the sequence. After the participant pressed the corresponding button, item 2 appeared to the right of item 1, and so on until the fourth response was made (**Figure 1A**). In the concurrent condition, fixation was followed by the appearance of all four items in the sequence (**Figure 1B**). All items remained on the screen until the fourth response was made thereby minimizing working memory demands. In both tasks, participants were given a maximum of 3000 ms to respond to all items. After the final response, a fixation cross appeared on the screen for the remainder of the trial. Total trial duration was 3333 ms. Syllable and word stimuli appeared in lowercase, white, bold, 35 point Arial font against a black background. In the baseline task, fixation was followed by the appearance of three line drawings (**Figure 1C**). After 300 ms, a yellow highlight appeared at random around one of the three drawings. Participants were given a maximum of 500 ms to respond. After the response, a fixation cross appeared on the screen for the remainder of the trial. Total trial duration was 1000 ms.

Each participant completed two runs (one syllable, one word). The order of runs varied across participants (eight syllable first, six word first). Each run contained two blocks each of the three tasks. The baseline blocks were always presented at the beginning and end of each run. Each block consisted of 18 trials. The consecutive (A) and concurrent (B) blocks were presented between the two baseline blocks in ABAB or BABA order counterbalanced across participants (seven consecutive first, seven concurrent first). Each block consisted of nine trials.

### Stimuli

Syllable stimuli (la, ma, na) consisted of a consonant and a vowel (CV). Word stimuli (log, map, net) were CVC. For each stimulus type, four item sequences were created out of the three items such that each item appeared at least once and one of the items appeared twice (e.g., ma la ma na). Immediate repetition of an item (e.g., ma ma la na) was not allowed. There were a total of 18 syllable and 18 word sequences. Each appeared between 1 and 4 times. The baseline stimuli were three abstract line drawings that were designed to minimize covert naming. Each of the six possible three-drawing sequences appeared between 4 to 7 times per run.

### fMRI Data Acquisition and Analysis

Structural and functional images were acquired using a 3T Siemens Trio scanner at the Center for Functional and Molecular Imaging at Georgetown University. Structural images were acquired using a sagittal T1-weighted MPRAGE sequence (TR = 1900 ms, TE = 2.52 ms, flip angle = 9°, TI = 900 ms, slice thickness = 1 mm). Functional images were acquired using an echo-planar imaging sequence (TR = 3000 ms, TE = 30 ms, flip angle = 90°, slice thickness = 3 mm).

Images were processed and analyzed using FSL (Jenkinson et al., 2012). Non-brain voxels were removed using BET. Images were motion-corrected using MCFLIRT, spatially smoothed using a Gaussian kernel (FWHM = 5 mm) and high-pass filtered (100 Hz). Statistical maps were normalized to MNI-152 space. Functional activation was analyzed using a general linear model that contained covariates of interest for each task (consecutive, concurrent and baseline) convolved with a standard hemodynamic response function.

For the whole-brain analysis, contrast images for Concurrent > Consecutive from each subject were entered into a random effects model and subjected to cluster-level familywise error correction within FSL’s feat. Suprathreshold voxels were identified using *Z* > 3.1 (*p* < 0.001). The resulting clusters were tested for significance (corrected cluster *p* < 0.05). Because we were interested in differences between the concurrent and consecutive conditions arising from differential activation rather than deactivation relative to baseline, we masked the thresholded image from the contrast of interest with positive voxels (*Z* > 0) from the orthogonal contrast of Critical > Baseline.

**FIGURE 2:**
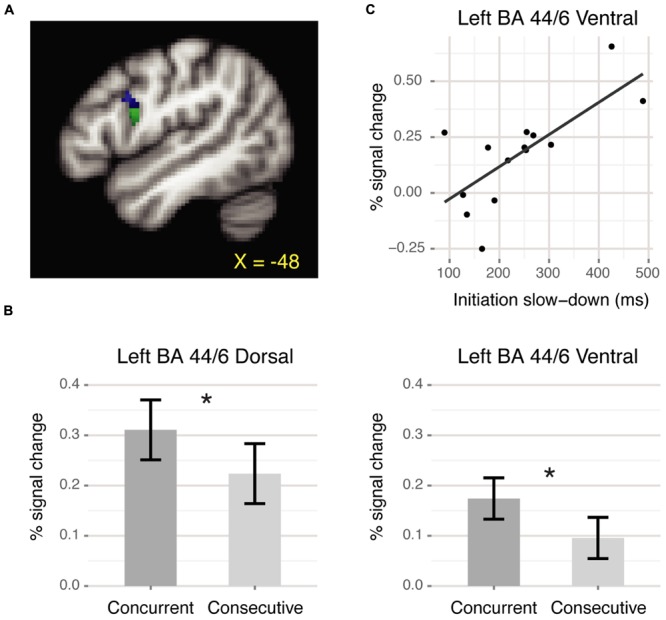
**BA 44/6 ROI results.** **(A)** Dorsal (blue) and ventral (green) ROIs at the premotor-prefrontal junction. **(B)** Mean percent signal change for each condition relative to baseline. Both ROIs showed significantly greater activation for the concurrent than the consecutive condition (^∗^*p* < 0.05). Error bars denote corrected Cousineau confidence intervals (Morey, 2008). **(C)** Positive correlation between slowed response initiation and neural activation within ventral BA 44/6 during the concurrent condition.

For the ROI analysis, we defined a left BA 44/6 region by masking positive voxels from the Critical > Baseline contrast with the intersection of left pars opercularis and left precentral gyrus (Harvard-Oxford structural atlas, probability >20). This region subsumed both ventral and dorsal portions of the premotor-prefrontal junction. Some researchers have suggested that the dorsal portion of the premotor-prefrontal junction, at the intersection of BA 44, 6 and 9, might be a distinct region within the frontal cortex (inferior frontal junction or IFJ: Brass et al., 2005; Derrfuss et al., 2012). Therefore, we split the region above into separate dorsal and ventral ROIs based on the intersection with the middle frontal gyrus from the Harvard-Oxford structural atlas (**Figure 2A**). The dorsal ROI comprised 82 voxels centered around (-49, 9, 29) and the ventral ROI comprised 171 voxels centered around (-53, 9, 18). Within each ROI, we compared activation for the concurrent and consecutive conditions using repeated-measures ANOVA over the mean percent signal change for each condition relative to baseline extracted via FSL’s featquery. Additionally, we examined the relation between left BA 44/6 activation and behavioral performance by computing correlations between activation within the two ROIs and reaction time (RT) measures for different participants. All correlations were computed using the skipped correlation technique and Spearman correlation, which is robust to outliers (Wilcox, 2005; Rousselet and Pernet, 2012. *scor* function in R available from http://dornsife.usc.edu/labs/rwilcox/software/). In these analyses, a *t*-value greater than the critical value indicates significance at α = 0.05.

To identify the functional networks involved in sequencing, we used generalized psychophysiological interaction (gPPI) analyses (McLaren et al., 2012). Mean time series for each left BA 44/6 ROI was extracted using fslmeants. The general linear model contained psychological regressors for each task, the physiological regressor, and interaction terms between each psychological regressor and the physiological regressor. A planned contrast of the concurrent–physiological interaction and the consecutive–physiological interaction was used to assess which regions showed increased functional connectivity with the seed region during the concurrent condition. The results were thresholded at *Z* > 2.3, cluster *p* < 0.05.

Given the well-known relevance of the left hemisphere for language and the locus indicated by prior studies with aphasic patients, we focused our analyses on left BA 44/6. To explore the extent of lateralization, if any, we also defined a right BA 44/6 region using the same procedures as above. We masked positive voxels from Critical > Baseline with the intersection of right pars opercularis and right precentral gyrus (Harvard-Oxford structural atlas, probability >20). The resulting region was split into dorsal and ventral ROIs based on the intersection with the middle frontal gyrus. The right BA 44/6 ROIs were smaller in extent than the corresponding left BA 44/6 ROIs. The dorsal ROI comprised 11 voxels centered around (52, 11, 24) and the ventral ROI comprised 75 voxels centered around (56, 10, 19). Within each ROI, we tested for Concurrent > Consecutive activation and correlation between neural activation and behavioral performance, as before.

Finally, to determine whether there were any differences between sequencing syllables and words, we tested for concurrent (syllables) > concurrent (words) and vice versa in whole-brain analysis and compared activation for these conditions within the left BA 44/6 ROIs.

## Results

### Behavioral Results

Mean accuracy for the three tasks was: consecutive = 84.13%, concurrent = 92.86%, baseline = 96.73%. Accuracy was higher in the concurrent than the consecutive condition [*F*(1,13) = 11.85, *p* < 0.005]. Consistent with previous research, in the concurrent condition relative to the consecutive condition, participants were slower to initiate responses [RT for item 1 = 956.58 ms (concurrent), 717.15 ms (consecutive). *F*(1,13) = 65.16, *p* < 0.001] but faster for subsequent items [RT for items 2–4 = 322.80 ms (concurrent), 493.62 ms (consecutive). *F*(1,13) = 129.60, *p* < 0.001]. Across subjects, the slow-down for item 1 was significantly correlated with the speed-up for subsequent items [*r* = -0.84, *t* = 5.34, *t*_crit_ = 2.82], suggesting that subjects who planned multiple responses ahead of time (resulting in slow initiation) benefitted later on (resulting in faster subsequent responses).

### Whole Brain Results

**FIGURE 3:**
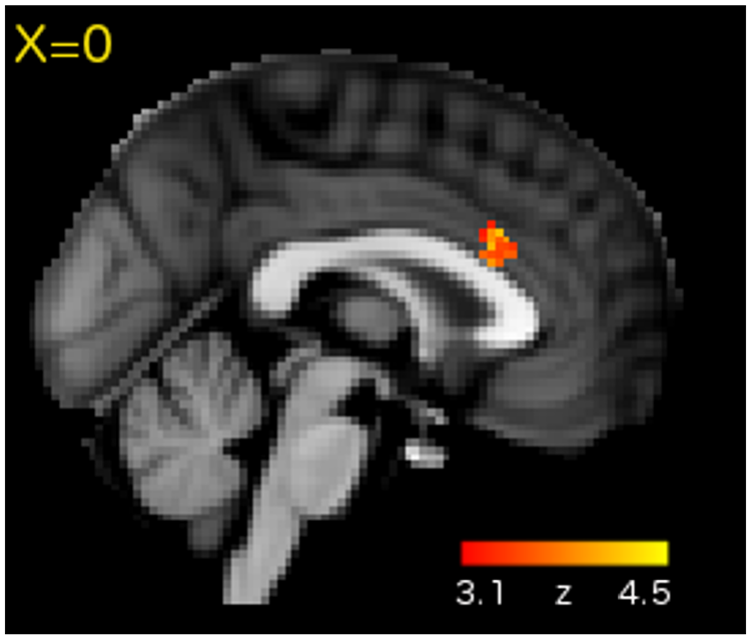
**Whole-brain analysis revealed Concurrent > Consecutive activation in the rostral portion of dorsal ACC**.

Regions showing significantly greater activation for the concurrent over the consecutive condition are shown in **Table 1.** Not surprisingly, there was increased activation in motor and somatosensory regions (pre and post-central gyri) associated with the execution of motor responses. We also detected effects in the visual and auditory cortices. Additionally, there was a significant effect in the rostral portion of the dorsal anterior cingulate cortex (ACC; **Figure 3**).

**Table 1:** Whole brain results.

# Voxels in cluster	Region	Max Z	*x*	*y*	*z*
227	Precentral gyrus/Postcentral gyrus (R)	4.13	44	-16	58
187	Superior parietal lobule/Postcentral gyrus (L)	4.23	-32	-40	58
124	Anterior cingulate gyrus	4.48	2	22	28
85	Superior parietal lobule/Postcentral gyrus (R)	4.34	22	-44	66
47	Callosal body (L)	3.93	-32	-42	0
41	Postcentral gyrus/Superior parietal lobule (R)	4.05	12	-46	60
30	Heschl’s gyrus (R)	4.05	44	-20	8
10	Temporal occipital cortex (L)	3.61	-38	-52	-2
9	Postcentral gyrus/Superior parietal lobule (R)	3.46	18	-44	72
5	Visual cortex (L)	4.1	-18	-64	-10
3	Precentral gyrus (R)	3.32	2	-20	54
3	Postcentral gyrus (L)	3.31	-46	-34	56
1	Postcentral gyrus (L)	3.23	-40	-38	62
1	Precentral gyrus (R)	3.19	14	-24	58
1	Parietal operculum (R)	3.13	38	-20	20


*Regions showing greater activation for the concurrent than the consecutive condition. Regions were identified by masking the Concurrent > Consecutive contrast (Z > 3.1, cluster p < 0.05) with positive voxels from the orthogonal Critical > Baseline contrast. The resulting voxels are those that surpassed thresholding for the contrast of interest and showed activation rather than deactivation relative to baseline. L = Left, R = Right.*

### ROI Results

Within the left BA 44/6 dorsal and ventral ROIs, there was significantly greater activation for the concurrent than the consecutive condition [Dorsal: *F*(1,13) = 4.99, *p* < 0.05; Ventral: *F*(1,13) = 8.52, *p* < 0.05, **Figure 2B**]. Additionally, in the ventral BA 44/6 ROI there was a significant correlation between the behavioral measure of slowed response initiation and neural activation for the concurrent condition [*r* = 0.66, *t* = 3.02, *t*_crit_ = 2.82, **Figure 2C**]. Those who were slower to initiate responses during the concurrent than the consecutive condition, suggestive of planning ahead, activated ventral BA 44/6 more. The corresponding correlation for the dorsal BA 44/6 ROI was not significant [*r* = 0.54, *t* = 2.24, *t*_crit_ = 2.82].

### PPI Results

**FIGURE 4:**
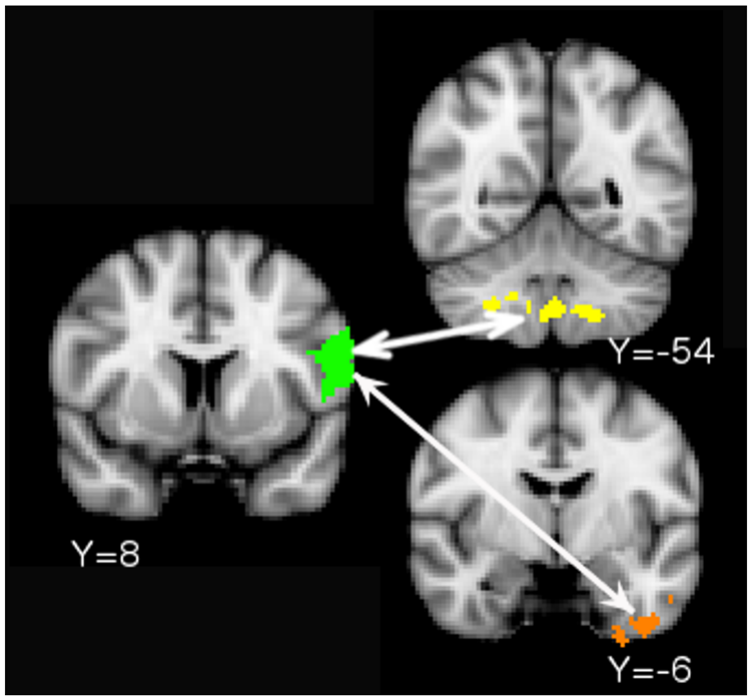
**Psychophysiological interaction (PPI) results.** Increased functional connectivity between the left BA 44/6 Ventral ROI (seed in green) and cerebellum/brain stem (yellow. Cerebellum peak: 26 -54 -44) and left anterior-inferior temporal lobe (orange. Peak: -28 -6 -8) during the concurrent task relative to the consecutive task.

The PPI analysis detected increased functional connectivity between the left BA 44/6 ventral ROI and (i) the posterior lobe of the cerebellum and (ii) the anterior portion of the left inferior temporal lobe during the concurrent versus the consecutive condition (**Figure 4**). A similar analysis for the left BA 44/6 dorsal ROI did not yield any significant clusters.

### Lateralization Results

Neither ROI in the right hemisphere showed significantly greater activation for the concurrent than the consecutive condition [Dorsal: *F*(1,13) = 2.49, *p* > 0.1; Ventral: *F*(1,13) = 0.23, *p* > 0.6]. There was no correlation between activation for the concurrent condition in these regions and slowed response initiation [Dorsal: *r* = 0.16, *t* = 0.55. Ventral: *r* = 0.09, *t* = 0.31, *t*_crit_ = 2.82]. These results differ from those for the left BA 44/6 ROIs. We tested whether activation for the concurrent condition was greater in the left than in the right hemisphere. The results suggest greater recruitment of left than right BA 44/6, with a significant difference in the comparison of the dorsal ROIs and a marginally significant difference in the comparison of the ventral ROIs [Dorsal left > right: Concurrent (left) = 0.31, Concurrent (right) = 0.09, *t*(13) = 1.91, one-tailed *p* < 0.05; Ventral left > right: Concurrent (left) = 0.17, Concurrent (right) = 0.06, *t*(13) = 1.71, one-tailed *p* = 0.06].

### Syllables vs. Words Results

Whole brain analysis revealed greater activation during concurrent (syllables) than concurrent (words) in a single cluster encompassing the anterior lobe of the cerebellum (Peak: 10 -42 -14) and nearby deep cortical structures (Peak: 18 -24 -16). The reverse contrast (words > syllables) did not reveal any clusters. Within the left BA 44/6 ROIs, we did not detect any significant differences between activation for syllables and words [Dorsal: *F*(1,13) = 1.5, *p* > 0.2; Ventral: *F*(1,13) = 1.43, *p* > 0.2].

## Discussion

We examined the neural correlates of sequencing with a focus on the left premotor-prefrontal junction (BA 44/6), which has been implicated in this function by previous studies. Participants reproduced visually presented sequences of syllables and words using manual key presses. The items in the sequence were presented either consecutively or concurrently. We recorded participants’ behavioral responses and compared activation for the concurrent versus the consecutive condition in the whole brain as well as within regions of interest in BA 44/6. The results show that the concurrent task induced planning and recruited sensory, somatosensory and motor regions as well as areas involved in controlled processing. Below, we summarize the results and discuss the neural substrates before turning to an analysis of the control processes involved in the task.

### Neural Substrates

Behavioral analyses indicated that concurrent presentation of items encouraged planning ahead. Participants were slower to initiate responses during the concurrent than the consecutive condition. Such planning ahead resulted in a benefit when executing later responses. Participants were faster to respond to non-initial items during the concurrent than the consecutive condition. Further, the two behavioral measures were correlated—slower sequence initiation corresponded with greater subsequent speeding-up. Together, these results corroborate previous evidence by showing that the concurrent task triggered the concurrent planning and sequencing of multiple responses (Lepage and Richer, 1996; Thothathiri et al., 2012).

Whole-brain analysis revealed increased activation within visual, auditory, motor, somatosensory and cingulate cortices when participants sequenced concurrently presented items. Activation in the visual cortex is likely related to sensory processing of the stimuli, which were presented visually. Auditory cortex activation is routinely observed during speech motor sequencing, where it is thought to provide auditory feedback for tuning language production (Bohland and Guenther, 2006; Guenther, 2006). The critical tasks used in the current study did not require overt or covert speech. However, it is possible that subjects used sub-vocal rehearsal of the linguistic stimuli to complete the sequencing task and that this led to the recruitment of the auditory cortex. As expected, there was increased activation within pre- and post-central gyri, which are part of a somatomotor network associated with motor planning, motor execution and somatosensory feedback (Guenther, 2006; Yeo et al., 2011). Post-central gyrus activation extended to the superior parietal lobule. This region is associated with visuospatial attention (see e.g., Esterman et al., 2009) as well as more abstract shifts of mental attention (e.g., switching between categories during a verbal fluency task: Gurd et al., 2002). In the current study, performing the concurrent task might have involved selectively attending to individual items at the sensory and/or more abstract cognitive levels.

In addition to sensory, motor and somatosensory regions, the concurrent task also recruited the rostral part of dorsal ACC. The ACC is a functionally heterogeneous region associated with three key functions: motor planning, cognitive control, and affective processing (Paus, 2001; Margulies et al., 2007). Of these possibilities, the observed location within the rostral portion of dorsal ACC (**Figure 3**) is most consistent with a cognitive control function. Human and non-human morphometric and functional studies suggest division of the ACC along the caudal-rostral axis with the caudal aspect (close to the anterior commissure) being involved in motor planning and the rostral aspect (*y* plane > 10) being involved in more complex cognitive processes (Paus, 2001; Margulies et al., 2007). Rostral-dorsal ACC has been repeatedly linked to conflict detection and monitoring in different contexts, including cases where a response must be selected out of multiple possible options (Botvinick et al., 2004). Such a cognitive control function would be particularly useful during our concurrent sequence reproduction task, where the co-activation of multiple items and/or responses would have necessitated selection of the correct item and/or response for the correct position in the sequence.

Region of interest analyses revealed a significant difference in activation for the concurrent and the consecutive conditions within dorsal and ventral left BA 44/6. Activation for the concurrent condition within ventral BA 44/6 also correlated with a behavioral measure of sequence planning. These results corroborate previous evidence for left BA 44/6 involvement in sequencing (e.g., Gelfand and Bookheimer, 2003; Thothathiri et al., 2010, 2012). Previous research suggests that the frontal lobe’s involvement in sequencing is related to higher-level operations rather than the storage of specific content (Zanini et al., 2002; Papeo et al., 2015). The present finding of Concurrent > Consecutive activation is consistent with this account because the two conditions involved the same content (syllables/words) but differed in the operations that were required to be performed over that content.

Psychophysiological interaction analysis showed increased connectivity between ventral left BA 44/6 and the posterior lobe of the cerebellum. The posterior lobe of the cerebellum receives projections from the association cortex and is activated during complex cognitive tasks (Stoodley and Schmahmann, 2010). The locus of the functional connectivity results in the current study was in lobules VIII and IX. Gray matter reductions in these lobules have been noted in individuals with autism spectrum disorder and attention deficit hyperactivity disorder (but interestingly not in individuals with dyslexia, a phonological disorder) (Stoodley, 2014). These lobules are functionally connected to cortical networks involved in cognitive control (Buckner et al., 2011). One recent study used Granger causality mapping to argue that the role of the cerebellum in cognitive control networks might be to monitor for errors and trigger activation of VLPFC to enable behavioral adjustment (Ide and Li, 2011). Increased correlation between VLPFC and cerebellum during the concurrent versus the consecutive condition in the present study is consistent with the idea that the cerebellum triggers VLPFC recruitment when there is increased possibility of errors due to conflict. The PPI analysis also showed increased connectivity between ventral BA 44/6 and the anterior-most part of the left inferior temporal lobe, which is part of the ventral stream for visual processing (see e.g., DiCarlo et al., 2012 for a review). Increased frontal-inferior temporal connectivity during the concurrent condition suggests that this task involved more controlled processing of visual (written) objects than the consecutive condition.

We split the left BA 44/6 region into separate dorsal and ventral ROIs because some researchers have suggested that the dorsal portion near the middle frontal gyrus (the IFJ) might be a distinct region from the ventral portion (VLPFC). We found increased activation for the concurrent over the consecutive condition in both IFJ and VLPFC but significant brain-behavior correlation and increased connectivity with the cerebellum and the inferior temporal lobe for the latter but not the former. The two regions could potentially play different roles during sequencing. For example, the IFJ could be involved in the activation of task-relevant representations (Brass et al., 2005) and the VLPFC in selection between alternatives (Thompson-Schill et al., 1997).

Comparison between the concurrent conditions containing syllables versus words showed no difference between the two stimulus types in left BA 44/6. Whole brain analysis indicated greater activation for syllables within the anterior lobe of the cerebellum. Anatomical and functional evidence suggests that the anterior lobe of the cerebellum is connected to sensorimotor cortices and is activated specifically in sensorimotor (cf. cognitive) tasks (Stoodley and Schmahmann, 2010). Thus, these findings suggest that the sequencing of syllables and words recruited largely similar neural substrates with some additional sensorimotor processing for syllables. We did not detect any increased activation for words over syllables, which suggests that the word stimuli did not automatically trigger semantic processing and recruit alternate pathways for sequencing.

Together, the whole brain, ROI and PPI analyses show that the concurrent task recruited neural substrates associated with controlled processing, specifically rostral-dorsal ACC, left BA 44/6, and frontal-posterior cerebellar connectivity, in addition to sensory, motor and somatosensory regions. Below we consider the different ways in which participants could have approached the concurrent task and the types of control processes that could be supported by the regions above.

### Control Components of the Concurrent Task

The concurrent task involved the same stimuli as the consecutive task but presented them simultaneously rather than one at a time. Although participants *could* approach the concurrent task in the same way as the consecutive task, processing items one at a time, evidence from this and previous studies shows that they resort instead to concurrent planning and sequencing. In the present paradigm, such sequencing could have occurred at the level of the stimulus items, at the level of the motor responses, or both. One possibility is that when presented with a stimulus like “ma la ma na”, participants encoded the sequence of items, retrieved each item in order, and converted each item to the corresponding motor response prior to execution. Alternatively, they could have first converted each item to its corresponding motor response, stored the sequence of motor responses, and then retrieved and executed each response in order. A third possibility is that participants stored and retrieved sequences of items as well as responses. All three possibilities require controlled processing to store and retrieve representations in the correct order. An open question is whether this controlled processing operated solely at the sensory and motor levels or whether it also involved additional cognitive processing. The concurrent task used in the present study employed syllable and word stimuli but required only manual responses. Thus, it was possible for the participants to rely solely on the controlled processing of visual stimuli and motor responses and bypass the controlled processing of linguistic representations. The lateralization analysis found evidence for asymmetry between left and right BA 44/6. Specifically, there was no difference between the concurrent and consecutive conditions and no correlation with the behavioral measure of planning in the right BA 44/6 ROIs, unlike the effects in the left BA 44/6 ROIs. This tentatively suggests that participants accessed and processed the representations associated with the linguistic stimuli and did not rely solely on visuomotor mappings. However, interpretation of these lateralization effects is potentially confounded by the fact that our right-handed participants were asked to use the right thumb for two buttons versus the left thumb for one. Therefore, we interpret the Concurrent > Consecutive effects within the control networks conservatively, as reflecting controlled processing more broadly rather than higher-level cognitive control in particular.

A second open question that pertains to left BA 44/6 specifically is whether the controlled processing within this region should be characterized as a specialized sequencing function or whether such a function is a manifestation of broader selection under competition. Prior evidence predominantly favors the latter explanation for left VLPFC as a whole. This region has been implicated in tasks that manipulate selection demands but do not involve any sequencing (see e.g., Thompson-Schill et al., 1997). Similarly VLPFC-posterior cerebellar connectivity is modulated during non-sequencing tasks such as the stop signal task (Ide and Li, 2011). In the present study, we found activation within left BA 44/6 for the both the concurrent and the consecutive tasks relative the baseline. Because the consecutive task did not involve sequencing, this suggests that left BA 44/6 was involved in a process that was engaged during both tasks, only more so during the concurrent task. In our analysis, there are two possible interpretations that are consistent with this pattern. Left BA 44/6, much like left VLPFC as a whole, could support selection between competing alternatives, or it could support controlled phonological processing (see Thothathiri et al. (2012) for a detailed discussion). Future studies that employ phonological and non-phonological stimuli and manipulate selection demands in sequencing and non-sequencing contexts could inform this issue.

### Final Remarks

In this study, we examined the neural correlates of sequencing using a sequence reproduction task that has been used before in patients with language production difficulties. We used a simpler task than language production and manual rather than oral responses in order to isolate the sequencing component from semantic and syntactic processes and circumvent head motion related artifacts. That said, although the task most closely mimics word- and non-word repetition (phonological input followed by motor output), we believe that the results have broader implications for understanding language production in healthy adults and patients with aphasia. Speakers are known to plan utterances multiple words at a time rather than incrementally. 3Even though the consecutive condition did not present items simultaneously, items were presented and responses were executed close in time and the task was not highly practiced. Under these conditions, one might expect competition between previous and current items or responses and the involvement of selection processes. These co-activated words must be ordered prior to articulation just as co-activated representations must be ordered prior to manual sequencing in the current study. While questions about whether there is sub-specialization within the identified regions depending on stimulus type and response modality must await future research, this study demonstrates a role for left BA 44/6, rostral-dorsal ACC, and frontal-posterior cerebellar connectivity during temporal sequencing. More broadly, we suggest that although control is most often needed and studied under non-routine circumstances, it may also be needed for routine behaviors like sequencing that involve selection from amongst alternatives.

## Conflict of Interest Statement

The authors declare that the research was conducted in the absence of any commercial or financial relationships that could be construed as a potential conflict of interest.

## Footnotes

1 As these examples indicate, we focus here on sequence production and not sequence perception. The two functions might share some mechanisms but not others (e.g., change detection might be more important during perception than production).

2 Throughout this paper, we use “controlled processing” as an umbrella term to refer to a cluster of frontally mediated executive functions, including selection between alternatives, conflict monitoring and conflict resolution. Where appropriate, we use more specific terms to refer to specific sub-components.
